# Polyphenols: Benefits to the Cardiovascular System in Health and in Aging

**DOI:** 10.3390/nu5103779

**Published:** 2013-09-26

**Authors:** Sandhya Khurana, Krishnan Venkataraman, Amanda Hollingsworth, Matthew Piche, T. C. Tai

**Affiliations:** 1 Medical Sciences Division, Northern Ontario School of Medicine, Sudbury, ON P3E 2C6, Canada; E-Mails: skhurana@nosm.ca (S.K.); am_hollingsworth@laurentian.ca (A.H.); matthew.piche@nosm.ca (M.P.); 2 Department of Gerontology, Huntington University, Sudbury, ON P3E 2C6, Canada; E-Mail: kvenkataraman@huntingtonu.ca; 3 Department of Biology, Laurentian University, Sudbury, ON P3E 2C6, Canada; 4 Department of Chemistry and Biochemistry, Laurentian University, Sudbury, ON P3E 2C6, Canada; 5 Biomolecular Sciences Program, Laurentian University, Sudbury, ON P3E 2C6, Canada

**Keywords:** cardiovascular, ROS, polyphenols, aging, resveratrol, EGCG, curcumin, olive oil, quercetin, berries

## Abstract

Numerous studies have demonstrated the importance of naturally occurring dietary polyphenols in promoting cardiovascular health and emphasized the significant role these compounds play in limiting the effects of cellular aging. Polyphenols such as resveratrol, epigallocatechin gallate (EGCG), and curcumin have been acknowledged for having beneficial effects on cardiovascular health, while some have also been shown to be protective in aging. This review highlights the literature surrounding this topic on the prominently studied and documented polyphenols as pertaining to cardiovascular health and aging.

## 1. Introduction

### 1.1. Consequences of Diet on Health

The consequences of nutrition on health and well-being are quite well established. In fact, a popular saying that “you are what you eat” emerged from nutritionist Henry Lindlahr’s observations of a link between a healthy diet and better health, which was written in his book titled “You Are What You Eat: how to win and keep health with foods” (originally published in 1942) [1]. A quest to understand how increased consumption of certain foods leads to better health has generated an interest in polyphenols, natural compounds that are found in several edibles. Polyphenols are synthesized by plants as secondary metabolites and are usually synthesized as defense mechanisms against stressors such as pathogens [2]. Based on the number of phenolic rings as well as the structural moiety that holds these together, polyphenols are classified into four categories: phenolic acids, flavonoids, stilbenes and lignans, with the flavonoids further classified into six subclasses (flavonols, flavones, isoflavones, flavanones, anthocyanidins and flavonols) [3]. From two decades, these compounds have been extensively researched for their capacity to improve human health. These analyses include a wide variety of clinical and nutritional epidemiological studies that indicate that populations whose diets are rich in polyphenols are less susceptible to cardiovascular diseases along with their complications and related mortality [4,5]. The French diet with copious amounts of red wine, the culture of green tea consumption in far eastern diets, the centrality of turmeric in South Asian diet and the Mediterranean diet rich in olive oil allow for cuisines that are all dense in polyphenols [4,5,6,7].

### 1.2. Reactive Oxygen Species in Cardiovascular Diseases: Polyphenols as Potential Therapeutics

#### 1.2.1. Role of Reactive Oxygen Species in Disease

Numerous studies have supported a role for oxidative stress in the development and pathogenesis of a wide variety of diseases such as diabetes, Alzheimer’s disease, chronic lung disease and cardiovascular diseases; the principal contributor to oxidative stress in the body being the generation of excess reactive oxygen species (ROS) [8,9]. Typically, ROS production occurs during physiological processes like respiration and metabolism and is usually well regulated and monitored by the cellular defense mechanisms such as superoxide dismutase (SOD), catalase (CAT) and glutathione reductase (GSR) [10]. Under situations of stress, exposure to environmental pollution and in aging for example, ROS levels increase and the cell’s antioxidant system may be overwhelmed with excessive ROS, thus becoming deleterious to cell health and integrity. ROS are free radicals and highly reactive oxidizers that can bind DNA, lipids and proteins to attain stability, thereby turning a physiological condition into a pathological state [11]. ROS have been implicated in the development of diabetes, cardiovascular disorders and a variety of age-associated disorders like Parkinson’s and Alzheimer’s disease [10,11,12,13].

#### 1.2.2. Polyphenols as Potential Therapeutics for Cardiovascular Diseases

In the case of cardiovascular disorders, oxidative stress and ROS have been vastly implicated in endothelial damage, progression to atherosclerosis, and injury in sustained myocardial infarction, as well as in ischemia reperfusion [8,12,14]. A deterioration in nitric oxide (NO) dependent vasorelaxation is a well-established risk factor that can predispose individuals to cardiovascular disease, and has been accepted as a feature with tremendous value in the prognosis of cardiovascular health [15]. A decreased NO bioavailability can occur due to reduction in expression of endothelial NOS (eNOS), the enzyme that is responsible for NO biosynthesis in the endothelium, as well as reduction in the available NO due to degradation by ROS amongst other reasons [15]. The sources of ROS in the vasculature are many, with mitochondrial enzymes NADH/NADPH oxidase, xanthine oxidase and others being significant culprits [15,16].

The oxidation of LDL and thereafter its entry across the endothelial barrier is the initiating factor in the generation of atherosclerotic plaques. Further, the interplay between hypercholesterolemia, oxidative stress radicals and inflammatory molecules generates an environment prone to massive endothelial damage, a hallmark of atherosclerotic progression [17]. Vascular endothelial adhesion molecule-1 (VCAM-1), intercellular cell adhesion molecule-1 (ICAM-1) and *E*-selectin are membrane proteins that facilitate the adhesion of leukocytes to the vascular endothelium and atherosclerotic lesions thereby stimulating signal transduction cascades [17]. These pathways lead to infiltration of leukocytes and macrophages into atherosclerotic plaques and ultimately the release of proinflammatory cytokines like tumor necrosis factor α (TNFα), interferon γ (IFNγ) and migratory factors such as monocyte chemoattractant protein-1 (MCP-1). Eventually upon upregulation of the key transcription factor NFκB, interleukins (IL-6 and IL-8) and gelatinolytic enzymes like metalloproteinases (MMPs) and others are synthesized, all known to play a role in atherosclerosis development [18]. ROS-mediated early events in atherosclerosis also includes activation of platelets by the endothelial adhesion molecule P-selectin, followed by upregulation of thromboxane (TX) A2 and platelet derived factors such as CD40 [19]. Upon atherosclerotic plaque rupture, platelets can bind to the endothelium leading to tethering, aggregation, and thrombus formation, ultimately to embolism and vasoconstriction, both hallmarks of myocardial infarction [20]. Moreover, the migration and proliferation of vascular smooth muscle cells (VSMCs) at an exaggerated proliferative rate and migration into the intima are both critical factors in the pathogenesis of atherosclerosis [21].

Diseases such as hypertension, pressure overload and vascular stenosis can lead to structural changes in the heart such as hypertrophy which are exemplified by increases in ROS, potent vasoconstrictor molecules like endothelin-1 (ET-1) and angiotensin II (AngII), and activation of signaling pathways activated via MAP38 kinases and NFκB [22]. ET-1 has been implicated in the pathogenesis of ROS mediated vascular abnormalities including proliferation and hypertrophy in VSMCs by ROS mediated activation of protein kinase B (PKB), extracellular signal-regulated kinase 1/2 (ERK1/2) and protein tyrosine kinase (Pyk2) signaling [23]. ET-1 is found to be elevated in patients with hypertension [24]. Ang II, the fundamental determinant in the renin-angiotensin system is formed by the action of angiotensin converting enzyme (ACE) on angiotensin I, and is a crucial factor in the etiology of hypertension and resultant changes in cardiac morphology and remodeling [25].

Lastly, I/R injuries encountered in the heart, consequently leading to arrhythmias, cardiac stunning, microvascular damage and cardiac cell death by apoptosis, is more damaging since several ROS are increased in the heart following reperfusion [8,26]. Also, ischemic heart disease is accompanied by myocardial infarction leading to myocardial hypoxia, generation of ROS, and accumulation of waste metabolites which ultimately leads to cell death and atherosclerotic tissue [8]. Sirtuins, critical factors in cell division, aging and response to stress, especially SIRT1, is found to be dysregulated during hypertrophy and myocardial stress in the heart, is also a new target identified in the pathogenesis of CVD [27].

Antioxidant therapies have been gaining recognition as strategies to reduce ROS in the vasculature thereby diminishing their detrimental effects [9]. Inhibitors of ACE that reduce circulating AngII, have been shown to reduce oxidative stress in addition to their antihypertensive properties; statins have been employed for the same purpose in addition to their cholesterol reducing properties by virtue of modulating HMG CoA reductase and lastly, vitamin E and C have been used extensively as dietary aids in conjunction with other drugs to reduce oxidative stress [9]. Polyphenols on the other hand are beginning to gain recognition and acceptance as potential therapeutic agents that could be beneficial in combating oxidative stress and thereby protect individuals from cardiovascular diseases [28,29]. Historically, the beneficial effects of polyphenols have been attributed primarily to their antioxidant capacity and their ability to modulate cellular antioxidant defense mechanisms by inducing the synthesis of detoxification enzymes like SOD, CAT, glutathione *S*-transferase (GST), glutathione peroxidase (GPx), NAD(P)H quinone oxidoreductase1 (NQO1) amongst others [30,31,32]. However, recent research provides evidence of polyphenols as modulators of signaling pathways [33,34,35,36]. A variety of studies encompassing clinical trials, epidemiological data as well as *in vitro* and *in vivo* studies with animals have been performed to firstly establish a cause and effect link between a diet rich in polyphenols and improvement in health, and secondly to gain insight into the mechanisms of the mode of action and protection bestowed by these compounds [4,35,36,37]. A significant mechanism to prevent the development of atherosclerosis is to protect the endothelium, reduce the oxidation of LDL, reduce cholesterol levels and repress the synthesis of proinflammatory cytokines and adhesion molecules [2,6]. In this scenario, polyphenols have been shown to modulate a variety of targets which include eNOS and NO, inflammatory cytokines like TNFα, IL-6 and IL-8 in addition to VCAM-1 and ICAM-1, and modulating signaling pathways by altering SIRT1, MAP38 kinase, NFκB, AP-1 amongst many others [2,33,38,39,40,41].

### 1.3. An Aging Cardiovascular System—Role of ROS

As mammalian cardiovascular systems age, there are several changes in morphology, anatomy, physiology and biochemistry of the heart and associated vessels. Morphologically, the heart undergoes thickening of the left ventricle and hypertrophy of the left ventricle and interventricular septum. There is stiffening, scarring and calcification of aortic valve leaflets and aortic sclerosis. Mitral annular calcification (MAC) and apoptotic reduction of the SA and AV node’s pacemaker cells along with deposition of collagen, adipose tissue and amyloid occurs, changes electrical activity over the myocardium [42,43].

From the perspective of cardiac tissues, cardiomyocyte dimensions increase with an actual decrease in cell numbers. The sympathetic nerve supply decreases, resulting in reduced responsiveness to the beta-adrenergic pathway. Aging arteries are thickened, with the changes primarily in the intima and media and the sub-endothelial space may contain exaggerated deposits of collagen, elastin and proteoglycans [42,43]. VSMCs in the tunica intima are rounded with larger amounts of organelles [44]. Often, smooth muscle cells, macrophages and leukocytes migrate to the sub-endothelial space and are associated with increased levels of proinflammatory cytokines [44,45,46]. The consequent low-grade inflammation and endothelial damage is correlated with coronary artery disease and stroke in the elderly. The primary culprit for the considerable amount of tissue remodeling in the cardiovascular system is recognized to be ROS which have been implicated in both apoptosis and senescence of various cell types of the cardiovascular system [47].

ROS are generated primarily due to increased NAD(P)H oxidase activity and dysfunctional mitochondria [47,48]. A recent review by Ungvari and colleagues summarizes age related changes in signaling to the mitochondria, wherein reduced levels of NO, growth hormone (GH), insulin-like growth factor (IGF) and adiponectin combined with an increase in angiotensin II result in reduced mitochondrial turnover and biogenesis and an increase in ROS [48]. Mitochondria show reduced biogenesis and increased ROS production in aging cells and this impacts cells in multiple ways ranging from altered Ca^2+^ signaling, signaling stress induced protein kinases and TOR associated pathways. The increased ROS production is attributed to a combination of inactivation of MnSOD, cellular reduction of GSH levels, lower levels of Nrf2/ARE along with a dysfunctional electron transport chain [48].

The cellular targets for ROS in the aging cardiovascular system are many-fold. Targets include mitochondrial proteins and mitochondrial DNA amongst several other factors associated with apoptosis and inflammation. The impact of ROS on numerous targets manifests as inflammation, vascular rarefaction, and an increased rate of apoptosis in endothelial and smooth muscle cells. Ungvari and colleagues suggest that dysregulated mitochondrial turnover in an aging vasculature can contribute to an altered redox state in cells, leading to additional oxidative damage [48]. Targets identified thus far for ROS are transcription factors AP2, NFκB, Nrf2 and p53. Additionally, studies involving quenching of ROS with resveratrol, results in deacetylation and activation of PGC1α by SIRT1 and Nrf2, both of which are associated with mitochondrial biogenesis and mitigation of oxidative stress and redox homeostasis [48]. Additionally, ROS has also been linked with damage to essential factors in various fundamental cellular pathways such as glycolysis, nuclear transport, translation, proteasome function and chaperones [49].

### 1.4. The Focus of This Review

This review will highlight key studies and describe how polyphenols can counteract ROS as well as modulate signaling pathways to enhance health outcomes in the realm of cardiovascular disorders and aging. For the purpose of this discussion, this review will focus on evidence from literature supporting rescue from cardiovascular disease and aging as a result of oxidative damage, by dietary polyphenols. All the phenolics will be discussed with the objective of identifying studies that delineate their role in antioxidant defenses, anti-inflammation, VSMC proliferation and migration, anti-thrombolytic activity and finally protection in prevention or rejuvenation of damaged cardiac morphology as seen in ischemia reperfusion and hypertrophy in cardiac heart failure. The studies identified here are representative of broader findings that indicate the same biological phenomena.

The focus of this review is on food as natural sources of cardioprotective phenolic compounds, identifying the predominant phenolic compounds amongst the most potent foods that are acknowledged to be “superfoods” for the heart. Most of the well-studied phenolic compounds are polyphenolic in structure. The foods that comprise our categorization of “superfoods” are green tea, red wine, turmeric, capers, olive oil and berries. With the exception of olive oil and berries, the other “superfoods” have at least one well-studied polyphenol that they are abundant in, which have been highlighted here—resveratrol, EGCG, curcumin and quercetin, and will be discussed under bioactive components of polyphenol rich foods. As for olive oil, berries and fruits, which have been discussed in literature primarily as foods containing multiple phenolics, not necessarily all polyphenols, this review will address these foods and their effects based on findings using the whole food or extracts thereof as a collective of mixed phenolics.

## 2. Bioactive Components of Polyphenol Rich Foods

### 2.1 Resveratrol

#### 2.1.1 Dietary Sources of Resveratrol

Resveratrol is a stilbene compound, and a phytoalexin, synthesized by plants in response to stressful stimuli. In addition to its noteworthy and acclaimed presence in red wine, ports and sherries, resveratrol is found in red grapes, blueberries, peanuts, itadori tea, as well as hops, pistachios and in grape and cranberry juices [41,50]. The content of resveratrol varies depending on the source and processing of the fruit; for example, boiled peanuts have a much higher content (5.1 μg/g) than roasted ones (0.055 μg/g) while dried grapes skins have a much higher content (24.06 μg/g) than red grapes themselves (0.16–3.54 μg/g). Additionally, red grapes have much more resveratrol than white ones [50]. Also, resveratrol content in red wine is reported to be 0.1–14 mg/L while in white wines, it is <0.1–2.1 mg/L [41]. Furthermore the quantity of resveratrol in red wine varies depending on the type and the source of grapes used to produce the wine; the Pinot Noir has a higher quantity of resveratrol than a Merlot with an added difference in Pinot Noirs dependent on the origin of the grapes; Pinot Noir from Oregon being higher than the ones from Upstate New York [51]. According to Goncalves and Camara, employing a newer sophisticated method for quantification, the quantity of resveratrol can be up to 50 μg/mL in certain red wines which would translate to 15 mg in two glasses of red wine (300 mL) [52,53]. Tomato skin has also been found to have resveratrol, but the study also noted the differences in the content depending on the variety of the tomato, with the beefsteak having negligible resveratrol content compared to the MicroTom [54]. Recently, cocoa and dark chocolate were also identified as minor sources of resveratrol [55].

#### 2.1.2. Epidemiological Data and Clinical Studies on Resveratrol

The World Health Organization’s MONItoring trends and determinants in CArdiovascular diseases (MONICA) study collected data on food intake and parameters of cardiovascular health from individuals of 26 countries [56]. Some of the findings of the MONICA study suggested an inverse correlation of dietary fat intake and the risk of cardiovascular diseases attributed to the consumption of wine in French and Swiss populations; however in populations of the US, UK and Australia that consumed similar amounts of dietary fat but not an equivalent amount of wine, the rate of mortality from cardiovascular diseases was much higher [5]. The findings suggested that the detrimental effects of a fat rich diet were counteracted by the high consumption of wine, often referred to as the “French paradox”. Since then, resveratrol, the primary polyphenol found in red wine has come into the limelight, particularly to study its effects on risk parameters that are considered hallmarks of cardiovascular and other diseases. Numerous clinical trials have been undertaken to assess the correlation between wine consumption/resveratrol intake and the risk parameters for cardiovascular diseases [57]. Recent clinical trials in patients with stable coronary artery disease (CAD) investigated the effect of a daily oral intake of 10 mg resveratrol capsule for 3 months. The results showed a drastic improvement in flow-mediated vasodilation (FMD), which is dependent on vasodilation and is an indicator of endothelial function. Further, Low Density Lipoprotein (LDL) as well as platelet aggregation was significantly reduced in these patients [58]. In another more recent study, the effect of grape extract intake was analyzed in CAD patients. Resveratrol containing grape extracts decreased Apolipoprotein B and oxidized LDL, increased circulating serum adiponectin, an anti-inflammatory molecule also involved in lipid and glucose metabolism. Also plasminogen activator-1 (PA-1) levels that can be modulated by adiponectin were decreased in these CAD patients. Further, the production of proinflammatory cytokines like IL-6 and TNF-α were also reduced in PBMCs [59,60,61]. In another clinical trial evaluating the effect of resveratrol, exposure of endothelial cells to plasma obtained from healthy subjects consuming 400 mg/day of resveratrol for a month, demonstrated a reduction in the mRNA of inflammatory and adhesion molecules. The study noted a significant decrease in the expression of VCAM-1 and ICAM-1. The secretion of IL-8 was also repressed. All these inflammatory markers are directly correlated with atherosclerosis progression and their reduction demonstrates that consumption of resveratrol can be a preventative measure that can modulate plasma content and the onset of atherosclerosis [62].

#### 2.1.3. Mechanisms of Resveratrol Action—Cell and Animal Studies

Resveratrol has been extensively researched for its ability to modulate determinants that are linked with increased cardiovascular risk. Improvement in lipid profiles and cholesterol levels, reductions in blood pressure and platelet aggregation as well increase in lifespan have all been demonstrated with resveratrol intake [41,63]. Resveratrol can stimulate the activity of sirtuins, particularly SIRT1 a histone deacetylase which regulates expression of genes involved in the stress response and cellular aging as well as modulates the adenosine monophosphate kinase (AMPK) signaling pathway, that influences fatty acid and lipid metabolism [64,65]. Mitochondrial biogenesis and enhanced mitochondrial function has been demonstrated in endothelial cells treated with resveratrol, by inducing nuclear respiratory factor-1 (Nrf-1), mitochondrial transcription factor A (Tfam) and peroxisome proliferator activated receptor gamma co-activator 1α (PGC1α) [66]. In coronary arterial cells, resveratrol stimulated the activity of nuclear factor-2 (Nrf-2), a transcription factor which can bind to antioxidant response elements (ARE) and upregulate a variety of antioxidant enzymes such as NQO1, HO-1 and control γ-glutamylcysteine synthetase (GCLC), the enzyme that regulates glutathione synthesis [32].

In the endothelium, resveratrol can stimulate eNOS activity increasing the amount of NO, thereby increasing vasodilation in endothelial cells as well as in isolated rat aortas [67,68]. Resveratrol can also modulate the biosynthesis of thromboxanes TXA2 and TXB2, molecules involved in platelet aggregation, by inhibiting p38 MAP kinase and PKC activity thereby reducing platelet adhesion and aggregation, consequently being antithrombotic and atheroprotective [69,70,71]. It can also contribute to reduction in inflammatory markers by inhibition of NFκB, TNFα and IL-6 as well as ICAM-1 and VCAM-1 expression in coronary endothelial cells [72,73]. Also, it decreases STAT-1 activation thereby downregulating interferon-γ inducible genes in macrophages [74]. In C57BL/6J mice, a high fat diet renders these animals susceptible to dyslipidemia with increased cholesterol and oxidized LDL in the serum. Upon administration of 200 mg/kg diet/day resveratrol for 8 weeks, these parameters were reversed with a reduction in cholesterol and increased in HDL. This was mediated in part by the increase in cholesterol 7alpha hydroxylase (CYP7A1) in the liver, an enzyme that is important in bile synthesis and cholesterol homeostasis in the body [75].

A critical mechanism by which resveratrol can protect from cardiovascular diseases is by preventing structural changes in the heart precipitated by hypertension, pressure overload and vascular stenosis and preventing this hypertrophy and remodeling [41]. Numerous animal studies have also demonstrated that administration of resveratrol improves cardiac function and preserves cardiac anatomy. In the spontaneously hypertensive rat (SHR), resveratrol dose of 2.5 mg/kg/day for 10 weeks prevented cardiac concentric hypertrophy, improved systolic and diastolic function but without any change in blood pressure, as well as reduced arterial stiffening and increased compliance by inhibiting the ERK1/2 pathway thereby denoting that an intervention with resveratrol may prove beneficial in protecting from remodeling of heart tissue [76,77]. Further, this improvement in the SHR was demonstrated to be a consequence of increased serum NO due to an activation of the AMPK pathway in the heart tissue of these animals and an impediment in the norepinephrine induced hypertrophy in cardiomyocytes [78]. In another hypertensive model of heart failure, the Dahl salt sensitive (DSS) rats, a high salt diet induced high blood pressure, ventricular dysfunction and cardiac remodeling with loss in body mass and increased mortality. However, in the group that received 20 mg/kg/day of resveratrol, there was no reduction in the body weight with an improved survival rate of the animals fed a high salt diet [79]. The animals showed normal eNOS levels as compared to those high salt fed rats that didn’t receive resveratrol, with improved vasodilation and endothelial function. Moreover, resveratrol stimulated an upregulation of the expression of PPARα and its co-activator PGC1α, both involved in lipid metabolism [79]. Although there was no prevention of ventricular wall thickening as reported in the SHR model, this could be because of the differing antecedents of cardiac dysfunction in the different models. In Sprague Dawley rats, treatment with 5 mg/kg/day of resveratrol for 1 week prior and then after induction of myocardial infraction by ligation of the coronary artery, resveratrol suppressed the ventricular tachycardia and fibrillation by inhibiting l-type calcium current. Moreover, by week 14 resveratrol reduced infarct size by 20% and the overall mortality by 33% likely by suppressing remodeling of the left ventricle [80]. Resveratrol has been shown to be beneficial in protecting from damage caused by I/R injuries [41]. In neonatal cardiomyocytes, resveratrol can protect cells from I/R mediated cell apoptosis by reducing Bax and caspase3 expression and improving cell survival [81].

A more advanced and rapidly growing area of scientific exploration in understanding the molecular mechanisms involved in cardiovascular disease, is to comprehend the alteration of microRNA in disease and healthy states. MicroRNA are regulatory RNA sequences that can influence gene expression. For example, the microRNA profile of patients who suffer from coronary artery disease is markedly different from healthy subjects [82]. Therefore, the modulation of microRNA by resveratrol is an area of increasing interest and scientific research [83,84]. Specifically, an interesting study on global miRNA expression in hearts of rats that got exposed to ischemia followed by reperfusion, demonstrated that pretreatment of these animals with resveratrol or longivinex, a multicomponent resveratrol containing formulation, reestablished miRNA signatures to the same as baseline vehicle controls as compared to the miRNA expression patterns seen in the group that received no pretreatment [85]. This study emphasizes that resveratrol can modulate miRNA pathways and signaling mechanisms that might be critical in the cardioprotection offered by this polyphenol.

#### 2.1.4. Resveratrol in Cardiovascular Aging

Probably the most extensively studied polyphenol, proponents of resveratrol have ascribed longevity enhancing, anti-cancer as well as cardioprotective properties to it. All three properties may merely be a consequence of mitigation of oxidative stress [40,47]. The conundrum of low bioavailability of resveratrol and its rapid metabolism has prompted researchers to seek other stable analogs to understand the impact of this polyphenol. Regardless, it is widely accepted that resveratrol lowers lipid peroxidation and increases plasma antioxidant capacity directly or indirectly. Additionally, elevated plasma levels of resveratrol mimic effects of caloric restriction in older adults, the cardiovascular benefits of which are well documented. Resveratrol is a COX1 inhibitor. The selective inhibition of COX1 over COX2 results in reduced platelet aggregation and vasoconstriction. COX1 inhibition also translates to reduced endothelial inflammation [41]. Recent reports have additionally identified SIRT1 regulated genes as well as Nrf2 regulated genes to be modulated by resveratrol [47]. In a study on middle-aged mice fed a high calorie diet supplemented with resveratrol, the polyphenol was found to prevent the detrimental effects of the diet and alter the physiology of these animals to parallel those fed the standard diet. As the mice aged, resveratrol increased their lifespan, insulin sensitivity and modulated PGC1α and mitochondrial number [86]. Resveratrol is also a vasorelaxant as a consequence of being able to stimulate Ca^2+^ associated K^+^ channels and increasing NO signaling in the endothelium. It is not apparent if the antioxidant pathways of resveratrol are a consequence of direct binding of ROS or if it is directly involved in stimulating cellular antioxidant pathways. Regardless, there is evidence of favorable serum lipid profiles being induced by resveratrol. In rat models, resveratrol has been additionally demonstrated to protect against I/R injury [41]. Resveratrol has also been demonstrated to be anti-proliferatory, impacting migration of VSMCs and the remodeling of arterial walls, implicated in atherosclerotic lesions [87,88,89]. The age associated increases in synthesis of proinflammatory cytokines from arterial VSMC isolated from aged rhesus monkeys as compared to that from the young ones was dramatically reduced with resveratrol treatment, likely by inhibiting NFκB [90]. Briefly, resveratrol has been demonstrated to impact all the hallmarks of cardiovascular aging and disease and unquestionably has been the most studied polyphenol.

### 2.2 Epigallocatechin Gallate—EGCG

#### 2.2.1. Dietary Sources of EGCG

Epigallocatechin gallate (EGCG) is a polyphenol belonging to the catechin family, a group of polyphenolic compounds that are abundant within green tea [91]. Black and oolong teas also contain catechins including EGCG as well as epicatechin, epicatechin gallate and epigallocatechin. These three teas originate from the leaves of the *Camellia sinenis* plant but are distinct due to processing and fermentation. As a result of manufacturing, catechins encompass only 3%–10% of the solid content of black tea but 30%–42% of green tea [91]. Catechins are also found in a variety of food sources including fruits, vegetables, tea, wine, and chocolate among others; however, EGCG is almost exclusive to tea [92,93].

#### 2.2.2. Epidemiological Data and Clinical Studies on EGCG

There is evidence for tea polyphenols to be the bioactive components contributing to the beneficial health effects observed in tea drinkers. Consumption of catechin-rich tea positively affected body weight, body-mass-index (BMI), waist circumference, body fat mass and subcutaneous fat of individuals following a 12 week intervention period [94]. Epidemiological observations highlight the association between heavy tea consumption and the apparent risk reduction in cardiovascular disease [95]. For example, tea consumption was found to be inversely associated with mortality due to all causes, including cardiovascular disease, in a study of 40,000 Japanese individuals. Lower risk of mortality was observed with consumption of greater than five cups of green tea per day in comparison to less than one cup per day [95]. Similarly, risk of developing hypertension in a Chinese population drinking 120–599 mL of tea per day was 46% lower than occasional tea drinkers and 65% lower when tea was consumed at a rate of 600 mL or more per day [96]. Tea consumption within elderly populations, as observed in participants of the Zutphen Elderly Study and the Rotterdam Study, is inversely related to mortality from coronary heart disease and incidence of a first myocardial infarction, with the risk of coronary heart disease and myocardial infarction being lower in heavy tea drinkers [97,98,99]. The inverse association of green tea intake in these studies and the incidence of cardiovascular disease might be associated with the decrease in triglycerides, total and LDL cholesterol as well as HDL cholesterol increase observed in tea drinkers [100]. A compilation of epidemiological studies examining green tea intake and cardiovascular risk revealed that of the 30 studies examined, 17 showed significant benefits of green tea consumption, 11 indicated no effects and the remaining studies documented negative effects [101,102]. Studies revealing negative or no effects of tea intake might be due to lack of consideration of other lifestyle factors and diet and the addition of milk to tea which may diminish its protective effect. Furthermore, the kind, preparation and strength of tea might also contribute to the lack of cardioprotective effects [101,103,104].

#### 2.2.3. Mechanism of Action of EGCG—Cell and Animal Studies

The major tea polyphenol, EGCG, demonstrates beneficial cardiovascular effects, including: protection from endothelial dysfunction, hypertension, cardiac hypertrophy, cardiac cell damage and injury. Experimental studies demonstrate the anti-hypertensive effects of tea and its major catechin, EGCG. Blood pressure of SHRs was attenuated following black or green tea consumption likely through modulation of the production of eNOS and NO, increasing bioavailability of NO to improve endothelial dysfunction and mediate vasodilation [38,105]. EGCG treatment of human endothelial cell lines enhanced eNOS mRNA production and facilitated a 60% increase in NO levels [106]. Stimulation of NO production involves inhibition of p38 MAPK phosphorylation and a H_2_O_2_ mediated activation of fyn, a Src family kinase that activates PI3 kinase/Akt and ultimately eNOS synthesis [33,107,108]. EGCG stimulation of NO via PI3 kinase induction was also shown in a study involving isolated mesenteric vascular beds (MVB) from SHRs wherein, the vasodilatory effect of EGCG was abolished after treatment with both a NO synthase inhibitor (l-NAME) and a PI3 kinase inhibitor (wortmannin) confirming the mechanism of action for EGCG [109]. The activation of PI3 kinase and p38 MAPK by EGCG also results in the upregulation of Nrf2 and activation of ERK 1/2, leading to an increase in the synthesis of HO-1 which in turn provides protection from H_2_O_2_ mediated oxidative stress and restricts expression of VCAM-1 [110,111,112]. Experimental models of atherosclerosis display upregulated inflammatory markers, including C-reactive protein (CRP), which is present in atherosclerotic lesions. Expression of CRP, a reliable marker of inflammation, was lower in animals subjected to an atherogenic diet supplemented with EGCG [113].

Rats fed a high-cholesterol diet exhibit poor lipid profiles, including high levels of serum and hepatic cholesterol, LDL and triglycerides along with low levels of HDL. Recovery of these lipid parameters were observed with green tea supplementation [114,115]. EGCG appears to affect cholesterol synthesis via the inhibition of hydroxyl-3-methyl-glutaryl-CoA (HMGR) activity, the rate-limiting enzyme in cholesterol synthesis [116]. Further, consumption of tea containing 58% EGCG reduced fatty plaque coverage within the aorta of ApoE-deficient mice [114]. EGCG treatment inhibited PKC and ERK 1/2 signaling pathways subsequently suppressing proliferation of VSMCs exposed to high glucose; EGCG also limits the migration of VSMCs, by inhibiting the activity of MMP-2 and MMP-9 [117,118,119]. Specifically, EGCG inhibits the activity of MMP-2 by blocking the activation of its catalytic subunit to reduce its gelatinolytic activity [119,120]. Platelet aggregation is repressed by EGCG by inhibiting p38 MAPK and ERK 1/2 while stimulating tyrosine phosphorylation of Syk and the adaptor protein SLP-76, which are requirements for intracellular Ca^2+^ elevation and platelet aggregation via the PKC pathway [121]. Synthesis of TXA_2_, is also inhibited by EGCG [122]. Synthesis and secretion of ET-1 is regulated by the transcription factor, FOXO1, which binds to the promoter of ET-1 to facilitate activation. This process is impeded when FOXO1 is phosphorylated by AMP kinase and Akt rendering it incapable of activating ET-1. The inactivation of FOXO1 occurs through cytoplasmic targeting, upon treatment of endothelial cells with EGCG [123].

EGCG can attenuate cardiac hypertrophy, myocyte hypertrophy and fibrosis developing from aorta constriction. EGCG has been shown to further prevent increases in left ventricular dimensions as well as improve systolic dysfunction that is apparent in aortic-constricted animals [124]. Cardiac hypertrophy is accompanied by generation of ROS as is evident by increases in MDA and reductions of endogenous antioxidant enzymes including SOD and GPx; EGCG recovered these parameters indicating its potential role as an antioxidant to combat oxidative stress injuries related to cardiac hypertrophy [125]. In addition to cardiac hypertrophy, the progression of the pathophysiology of atherosclerosis via ROS production is significantly diminished with EGCG exposure. In response to oxidized LDL, EGCG exposure minimizes the activation of NFκB and p38 MAPK pathway via NADPH oxidase and Lox-1 expression [126]. Impairments of the coronary artery blood supply results in I/R injury, also accompanied by oxidative stress. Isolated rat hearts undergoing I/R injury exhibit increases in lipid peroxidation and decreases in mitochondrial and cytoplasmic SOD (Mn-SOD, Cu/Zn-SOD) and catalase levels [127]. Features characteristic of apoptosis were evident in perfused left ventricular tissue of male rats, including increases in cleaved caspase-3 and Bax, and decreases in Bcl-2 proteins. EGCG protected from subsequent apoptosis following I/R injury by regulating the Bcl-2/Bax ratio and blocking the cleavage of caspase-3 [127,128]. Moreover, in cardiac myocytes, EGCG impaired STAT-1 phosphorylation which is involved in I/R induced apoptosis thereby protecting from cell death and improving recovery [129]. EGCG perfusion increases coronary blood flow by means of decreasing LDH levels, reducing infarct size, and improving ventricular function [127,130]. Cardiac necrosis and stunning of the heart post-reperfusion were reduced when EGCG was administered at time of reperfusion [130]. An interesting recent study investigating the impact of EGCG on I/R injury revealed that higher doses did not equate to greater protection, indicating the importance of dosing to achieve optimal cardiovascular protection [128]. Injury to the myocardium following I/R is exemplified by neutrophil infiltration leading to an exaggerated oxidative stress response and an increase in infarct size, an indirect result of cardiac myocyte release of proinflammatory cytokines, such as TNFα and IL-6 [131]. EGCG reduced expression of IL-6 and TNFα in a model of I/R injury by inhibiting NFκB [132]. Additionally, EGCG has been shown to reduce the migration of neutrophils in a cell culture model [133,134]. Experimentally induced myocardial infarction leads to diminished endogenous antioxidants and antioxidant enzymes as well as increased plasma lipid peroxides and uric acid in animals; supplementation with EGCG reversed the effects thereby aiding in the protection against myocardial infarction injury [135].

The observations highlighted above illustrate the cardioprotective qualities of EGCG and suggests its potential use as a protective agent in the prevention and improvement of cardiovascular disease.

#### 2.2.4. EGCG in Cardiovascular Aging

The most abundant catechin in green tea is EGCG. EGCG has been found to exert cardioprotective effects in several studies [95,136,137,138]. In a longitudinal study in 2006, Kuriyama *et al.*, discovered that cardiovascular disease associated mortality was greatly reduced in older adults consuming five or more cups of green tea every day [95]. EGCG was found to reduce cardiomyocyte apoptosis by inhibiting telomere attrition [139] and attenuated left ventricular remodeling, probably by decreasing oxidative stress [124,140,141]. EGCG was also found to improve endothelial function in patients with cardiovascular disease [136]. Similar findings amongst different cohorts have been summarized in a recent review [142]. EGCG reduces inflammation by indirectly regulating angiotensin II and consequently, NFκB. Other targets impacted by EGCG in smooth muscle cells (SMC) include IκB kinase, cJun, AP1, FAS receptor, STAT1, catalase, HO-I, and Nrf2 in endothelial and SMC. EGCG treatment also impacts migration and proliferation of SMC, thereby reducing progression of artherosclerosis. The mechanism for inhibition is believed to be EGCG incorporation into various cell compartments. EGCG has been shown to cause a G1 arrest by inhibiting cyclins D1 and E. Additionally, EGCG has also been shown to inhibit PCNA. Platelet function is also modulated by EGCG through regulation of PDGF. PDGF regulates several mitogenic genes such as ERK1/2, cFOS and EGR1. Factors down regulated in the inflammation pathway include TNFα, IL-12p40, p38 MAPK. All of this downregulation is accomplished through ERK1/2 activation, which breaks down IκBα and NFκB activation [33]. Endothelial function is improved by modulation of eNOS. Vasorelaxation is seen in both an NO dependent and an NO independent manner accompanied by an increase in cGMP levels [136]. The P13-Akt pathway is implicated along with estrogen receptor (ER) mediated pathways but no receptor for tea catechins or polyphenols have been identified in the cardiovascular system. Moreover, dietary intervention of aged Fischer 344 rats with EGCG was also found to lower the age-related oxidative damage in these animals by reducing oxidative stress, maintaining mitochondrial integrity while significantly declining the plasma level of 8-hydroxy-2′ deoxyguanosine, a critical biomarker of oxidative stress [143]. Most of the evidence of EGCG’s antithrombotic activity comes from *in vitro* evidence. Multiple factors are attributed to the potential for EGCG as an anti-thrombolytic factor such as lower levels of platelet activation factor (PAF), the inhibition of related acetyl transferases, inhibition of thrombin induced phosphorylation of p38MAPK and ERK1/2, the inhibition of tyrosine phosphorylation of several platelet proteins, and reduction of intracellular Ca^2+^ level associated fibrinogen binding in platelets [33]. The impact of EGCG in mitigating cardiovascular disease development and in retardation of cardiovascular aging is unquestionable and its potential benefits as a component of daily diet cannot be overstated.

### 2.3 Curcumin

#### 2.3.1. Dietary Sources of Curcumin

The polyphenol, curcumin, is the active component of turmeric, a common Indian spice, derived from the rhizome of the *Curcuma longa* plant [144,145]. Curcumin is the most abundant constituent of turmeric; comprising approximately 2%–5% of the compound [146].

#### 2.3.2. Epidemiological Data and Clinical Studies on Curcumin

Clinical studies of volunteers on a week-long regimen of curcumin (500 mg) had decreased lipid peroxides and total serum cholesterol, and increased serum HDL levels [147]. Similarly, patients with acute coronary syndrome administered low doses of curcumin at 15 mg, three times daily for 2 months had greater reductions in LDL and greater elevations in HDL levels. However, triglyceride levels were higher compared to basal levels with the moderate dose of 30 mg, three times daily for 2 months, having a minimalist effect on increased levels [148].

#### 2.3.3. Mechanism of Action of Curcumin—Cell and Animal Studies

Turmeric extract can influence the characteristic hypercholesterolemic effects of atherosclerosis reproduced in experimental models [149]. Rabbits administered a low dose (1.66 mg/kg body weight) of turmeric extract had a decreased susceptibility of LDL peroxidation, decreased total plasma cholesterol including lowered levels of LDL, triglycerides and phospholipids [149]. Further studies involving *Curcuma longa* extract supplementation in rabbits consuming an atherogenic diet revealed lessened plasma lipid peroxidation and fewer fatty streak lesions in the aorta. Additionally, α-tocopherol levels were greater with curcumin supplementation indicating the enhancement of endogenous antioxidant mechanisms [150]. This study also demonstrated the importance of dosing in the effectiveness of curcumin to attenuate cardiovascular complications since higher doses were not as effective as lower doses [149,150]. In addition, curcumin treatment of LDL-receptor knockout mice (LDL-KO) fed a high cholesterol diet increased plasma HDL and decreased LDL. Further, atherosclerosis biomarkers including atherogenic index, percent HDL:total cholesterol and ApoB:ApoA-1 ratio improved with curcumin treatment [151]. Atherosclerotic lesions subject to infiltration of lipids as well as the presence of ICAM-1 and VCAM-1 within aortic fatty plaques were detected in untreated LDL-KO mice consuming a high cholesterol diet but absent in mice administered curcumin [151]. In an alternate model, consumption of a high fat diet in hamsters demonstrated lower levels of free fatty acid, total cholesterol, triglyceride and leptins when supplemented with curcumin as well as elevations of plasma of HDL, apolipoprotein and paraoxonase activity [152].

Endothelial dysfunction modeled in porcine coronary arteries was attenuated with curcumin addition through impeding the homocysteine-induced impairment of endothelial-dependent vasorelaxation and eNOS levels as well as reducing superoxide anion production [153]. Curcumin relaxed pre-constricted porcine coronary arterial rings only in rings with intact endothelium and not when an inhibitor of NO synthesis, l-NNA (*N*-nitro-l-arginine) was introduced [154]. Furthermore, curcumin reduced transcript levels of TNFα receptors, diminished nuclear translocation of NFκB affecting gene regulation, and reduced TNFα-induced expression of adhesion molecules characteristic of atherosclerosis such as ICAM-1, MCP-1 and IL-8 mRNA and protein, as well as monocyte adhesion to HUVECs [145]. Curcumin also attenuates phosphorylation of PKB and ERK1/2 in VSMCs as well as inhibited c-Raf and insulin-like growth factor type 1 receptor (IGF-1R), all necessary for ET-1 activation thereby showing potential to modulate ET-1 effects in vascular physiology [155].

Curcumin possesses antioxidant properties that protect against oxidative effects on proteins and lipids [156]. Intracellular ROS levels within TNFα-stimulated HUVECs were attenuated with curcumin treatment [145]. Antioxidant abilities of curcumin were demonstrated in rats undergoing Adriamycin-induced myocardial toxicity [157]. Increased levels of lipid peroxidation products and catalase activity, in addition to, decreased myocardial glutathione and GPx activity, accompanied myocardial toxicity in Adriamycin treated rats; the group receiving curcumin therapy did not present with ECG abnormalities seen with their Adriamycin-treated counterparts nor did they have increased serum lipid peroxides and lipid peroxidation products. Curcumin augmented the endogenous antioxidant systems as confirmed by increased glutathione levels, glutathione peroxidase activity and reduced catalase activity [157]. In addition, protection of aortic endothelial cells against H_2_O_2_ induced oxidative stress is mediated by a concentration- and time-dependent induction of HO-1 by curcumin, protection from cell injury and protein oxidation [158].

Remodeling of vasculature after injury can have pathogenic consequences leading to cardiovascular complications. Growth factors, including platelet-derived growth factor (PDGF), play an important role in vascular remodeling [159]. PDGF-stimulated VSMC migration, proliferation, cytoskeletal reorganization and collagen synthesis were inhibited with curcumin treatment. Mediation of remodeling events by curcumin is done through blockage of signaling events including: PDFGF receptor binding, increased phosphor-tyrosine levels on PDGF-receptor β, and phosphorylation of downstream effectors: Erk1/2 and Akt [159].

High cholesterol-induced VSMC proliferation was modeled via administration of Chol:MβCD, a water-soluble cholesterol, to primary rat VSMC. Chol:MβCD causes VSMC proliferation and downregulates caveolin-1, an important regulator of cell proliferation via MAPK signaling. Suppression of ERK signaling was observed following curcumin treatment and cell cycle arrest occurred at the G1/S phase to inhibit VSMC proliferation [160]. LOX-1, an oxidized LDL receptor, and angiotensin II type 1 receptor (AT1R) are involved in hypertrophy of cardiac myocytes [161]. AngII is thought to activate AT1R to upregulate LOX-1 and influence oxidative stress production via NADPH oxidase and NF-κB activation to ultimately influence cardiac myocyte hypertrophy. Curcumin treatment blocks AngII-stimulation of AT1R and LOX-1, ROS generation, upregulation of NADPH oxidase and expression of redox-sensitive, NFκB. Interestingly, curcumin treatment alone attenuated basal levels of ROS, NADPH oxidase and expression level of NF-κB [161].

Protective effects of curcumin have been demonstrated in a case of MI. Microarray analysis identified differential expression of genes in rats undergoing coronary artery ligation and those administered curcumin before surgery [162]. Specifically, differences were observed in expression of genes involved in cytokine-cytokine receptor interaction, focal adhesion, apoptosis and extracellular matrix (ECM) receptor interaction. Differential expression is important since cytokine-cytokine receptors affect heart failure, ECM components are elevated in atherosclerotic lesions and focal adhesions have a role in post-MI remodeling. Biomarkers for MI, creatine kinase and lactate dehydrogenase (LD), were also elevated in coronary artery ligated rats but reversed in rats administered curcumin prior to surgery [162].

In summary, curcumin, the polyphenol common to the Indian spice, turmeric, demonstrates beneficial health effects in the prevention of cardiovascular disorders as well as attenuating factors involved in the pathophysiology of cardiovascular disease. The protective effects of curcumin imply that supplementation within the diet can be beneficial for cardiovascular health. 

#### 2.3.4. Curcumin in Cardiovascular Aging

First identified as an anti-inflammatory agent in 1995, curcumin’s ability to suppress inflammation by regulation of multiple cytokines such as beta-site APP-cleaving enzyme 1 (BACE-1), C-reactive protein (CRP), connective tissue growth factor (CTGF), endothelial leukocyte adhesion molecule-1 (ELAM-1), histone acetyl transferase (HAT), hypoxia inducible factor (HIF), ICAM-1, lipid peroxidation (LPO), MMPs, NFκB, ornithine decarboxylase (ODC), signal transducers and activator of transcription protein (STAT), TNFα, VCAM-1, vascular endothelial growth factor (VEGF), amongst others is evidence of the considerable potential for this polyphenol as a cardioprotective agent. In an extensive review published by Aggarwal and Harikumar in 2009 [163], the authors identified multiple sources of research that demonstrated physiological phenomena attributed to the role of curcumin in regulating the aforementioned factors. Some notable cardioprotective features of curcumin are inhibition of high glucose-induced foam cell formation by inhibition of NFκB, the inhibition of induced migration, proliferation and collagen synthesis in cultured VSMCs, the prevention of isoproterenol-induced myocardial infarction, decrease in the LPO of liver microsomes and mitochondria, inhibition of LDL oxidation, reduction of oxidative stress and reduction of aortic fatty streaks. Additionally, curcumin has been shown to decrease the levels of O^2−^, XO, MPO and LPO in myocardium elevated the levels of GPx, SOD, CAT and GST, inhibits the development of atherosclerosis in ApoE/LDLR-DKO mice, attenuates global cardiac I/R injury; decreases myocardial MMP-9, IL-6, MCP-1, TNFα, decreases plasma IL-8, IL-10, and cardiac troponin 1 and decreased apoptosis in cardiomyocytes and myocardial Myeloperoxidase (MPO). A detailed list of targets for curcumin as well as an extensive list of effect of curcumin on neurodegenerative, cardiovascular, neoplastic, pulmonary, metabolic, and autoimmune diseases is well documented [163]. A recent study supports the role for curcumin as a novel supplemental therapy for treatment of vascular aging by attenuating arterial stiffening and endothelial dysfunction preferentially in older mice [164]. In another study on postmenopausal women, curcumin ingestion for 8 weeks improved endothelial function as measured by flow mediated dilation [165]. Also, curcumin has been shown to mitigate cardiotoxicity due to adriamycin based anti-cancer treatments. An increase in cellular GST and reduced peroxidation of lipids due to curcumin’s ability to scavenge ROS has been attributed to this phenomenon. In the case of older patients with a co-morbidity of diabetes and cardiomyopathy, curcumin down regulates NOS and NO production by chelating NO_2_, an intermediate in production of NO. Abnormal accumulation of various connective tissue constituents in aging endothelia is a consequence of unstable lysosomal membranes. Curcumin reportedly stabilizes lysosomal membranes and decreases the activity of lysosomal acid hydrolases. It has additionally been suggested that curcumin may modulate hypertrophy in the aging heart by inhibiting the Adenoviral transcription co-activator, p300 [166].

The curcumin rich spice turmeric, prominently used in Indian cooking, is a promising candidate to aid in the healthy aging of the cardiovascular system.

### 2.4 Quercetin

#### 2.4.1. Dietary Sources of Quercetin

Quercetin, a polyphenol belonging to the flavonoid group is found in a wide variety of fruits and vegetables. Apples and onions, having a concentration of 4.57 mg/100 g and 22 mg/100 g respectively, are the significant sources of this flavonoid in the Western diet [167]. In case of onions, it has been shown that the different colored onions, yellow, red, pink or white, have varied amounts of quercetin and that the storage temperature affects the amount of this phenolic [168]. The yellow Sweet Savannah onion had the highest amount of quercetin (286 mg/kg) while white onions had negligible amounts of quercetin [168]. Interestingly, the largest amount of quercetin is found in capers 233 mg/100 g, and it is also found in cocoa powder at 22 mg/100 g [167]. Broccoli and green and black tea are also sources of quercetin [167]. Plums also contain quercetin, it being the most prominent polyphenol accounting for about two-thirds of the polyphenolic content found in this fruit; the quantities vary based on the cultivar tested in the study by Mubarak *et al.* in 2012 [169]. The amount of quercetin in the 29 cultivars tested in Mubarak’s study ranged from 9 mg/kg to 239.8 mg/kg. Plums also contain the glycoside of quercetin called Rutin ranging from 9.5 g/kg to 63.9 mg/kg [169]. Quercetin is also found in mulberry leaves, a medicinal plant used in China and Japan to aid in blood pressure reduction [170].

#### 2.4.2. Epidemiological and Clinical studies with Quercetin

Intriguingly, a randomized, double-blind, placebo-controlled, crossover study involving human patients with stage 1 hypertension has shown that treatment with high dose quercetin led to a reduction in systolic, diastolic and mean arterial pressure, suggesting the potential for quercetin to be used therapeutically in the treatment of early stage hypertension. Subjects were given 730 mg/day of quercetin for 28 days with findings compared to placebo and those subjects who were stage 1 hypertensive displayed a significant reduction in their systolic and diastolic blood pressure by 7 ± 2 mmHg and 5 ± 2 mmHg respectively. This clearly suggests that quercetin may be a viable therapeutic option in early hypertension [171].

Moreover, quercetin was studied in an at-risk population of overweight or obese individuals aged 25–65 years of age with metabolic syndrome traits, for its effect on blood pressure, lipid metabolism, along with markers of oxidative stress, inflammation and body composition. This double-blind, placebo controlled cross-over trial randomized patients to 150 mg quercetin/day *versus* placebo for a 6-week treatment period followed by a 5 week wash-out period. This study revealed that quercetin significantly reduced systolic blood pressure in all subjects by 2–6 mmHg, by 2–9 mmHg in the hypertensive subgroup, and by 3–7 mmHg for those patients aged 25–50 years. Furthermore, quercetin treatment significantly reduced the concentration of oxidized LDL without any detrimental effect on serum electrolytes, hematology, or liver and kidney function [172]. Taken together, this clinical study suggests that quercetin supplementation at 150 mg/day may therapeutically alter systolic blood pressure and reduce the concentration of oxidized LDL in a patient profile that is significantly at risk for cardiovascular disease.

#### 2.4.3. Mechanism of Action of Quercetin—Cell and Animal Studies

Quercetin can act as an indirect antioxidant, increasing the activities of phase 2 antioxidant enzymes GST, HO-1, NQO1 in cardiac ventricular myocytes isolated from WKY rats [173]. Using human umbilical vein endothelial cells, Balasuriya and Rupasinghe (2012) studied the effects of apple peel extracts rich in flavonoids and quercetin metabolites on ACE inhibition [174]. Interestingly, their work demonstrated that the flavonoid-rich apple peel extract as well as two quercetin metabolites inhibited ACE significantly with the flavonoids potentially acting as competitive inhibitors of ACE; notably, quercetin-3-*O*-glucoside and qyercetin-3-*O*-glucuronic acid significantly inhibited ACE [174].

Quercetin is a readily available flavonoid that has been suggested to be of benefit in ameliorating cardiovascular health via eNOS upregulation, and the reduction of oxidative stress [175]. Notably, studies involving rat aortic ring segments have demonstrated that quercetin treatment for 30 min enhanced relaxation of these aortic rings by virtue of NOS and endothelium derived hyperpolarizing factor. Moreover, this group demonstrated that bovine aortic endothelial cells, when treated with quercetin, exhibited an increase in intracellular calcium, eNOS phosphorylation and subsequent increase in NO. Taken together, these results suggest that quercetin induced phosphorylation of eNOS can increase availability of NO, thereby inducing protective vascular effects [176]. *In vivo* studies using SHRs have looked at the effects of quercetin administration on mean arterial pressure (MAP), heart rate and baroreflex sensitivity. SHR and their normotensive counterparts, the Wistar-kyoto rats (WKY), were treated with 2, 10 or 25 mg/kg/day oral quercetin or saline for 7 days. Notably, doses of 10 and 25 mg/kg/day were found to decrease MAP in SHR to 163 ± 4 and 156 ± 5 as compared to 173 ± 6 respectively with no change in WKY samples. Moreover, the dose of 25 mg/kg/day was found to decrease serum oxidative stress in SHR samples. This suggests that oral quercetin intake may play a protective role in decreasing blood pressure, perhaps via a mechanism linked to oxidative stress [177]. As Perez-Vizcaino *et al.* (2009) point out, quercetin has been studied in multiple rat models of hypertension and has been shown to induce a progressive and sustained reduction in blood pressure independently of the renin-angiotensin, oxidative stress or nitric oxide status without any effect in normotensive controls [178].

Lectin-like oxidized receptor 1 (LOX-1) is a receptor for oxidized LDL; activation of LOX-1 results in the subsequent increased expression of inflammatory cytokines as well as the decrease in the release of NO thereby disrupting the proper functioning of the endothelium [179]. LOX-1 is upregulated in physiologic circumstances highly linked to atherosclerotic disease such as hypertension, hyperlipidemia and diabetes [179]. Utilizing an *in vitro* model with Chinese hamster ovary cells expressing LOX-1, apple polyphenols inhibited uptake of oxidized LDL by 88%. Furthermore, SHR-SP rats were given oligomeric procyanidins purified from apples. At termination, the mesenteric artery of the rats displayed a significant reduction in the amount of lipid deposits in the arterial wall even in the context of a high fat diet [179]. Supplementation of diet in WKY rats with 0.5% quercetin, for 2 weeks resulted in altered serum lipid profile with an increase in LDL and decrease in HDL [180]. In ApoE deficient mice, a diet supplemented with Mulberry leaves (1%) reduced atherosclerotic lesions in the aorta by 40% as well as demonstrating a lag time in the onset of LDL oxidation [181]. Quercetin is also anti-atherosclerotic by inhibiting platelet aggregation [182]. In bovine aortic endothelial cells, treatment with mulberry leave extract inhibited the TNFα mediated activation of NFκB thereby repressing the inflammatory response, as well inhibiting the expression of LOX-1 [183]. Moreover, rabbits fed a high cholesterol diet (1% cholesterol supplement) displayed significant increases in CRP, total cholesterol, triglycerides and LDL, fibrinogen, nitrite, nitrate levels and a reduction in HDL levels [184]. Notably, administration of both high and low doses of apple juice, 10 mL and 5 mL respectively, lead to a decrease in total cholesterol, triglycerides, CRP, fibrinogen, and factor VII. On the other hand, 10 mL of apple juice supplementation lead to a significant decrease in LDL levels and an increase in the protective HDL levels. Taken together, these data suggest that apple juice supplementation may have a protective effect on the blood lipid profile *in vivo* [184]. Histologically, those groups supplemented with apple juice displayed a significant reduction in atherosclerotic lesions of the coronary arteries when compared to the high cholesterol diet group [184].

Quercetin treatment has been shown to prevent morphological and functional changes within organ systems such as blood vessels, kidney and heart secondary to hypertension. Quercetin also diminished the production of ROS associated with hypertension in the aforementioned rat models of the disease [185]. In Sprague Dawley rats undergoing myocardial I/R via coronary artery occlusion, infarct size was significantly reduced with quercetin treatment and inflammation was prevented by reduction of TNFα and IL-10 [186].

#### 2.4.4. Quercetin in Cardiovascular Aging

Quercetin is found in several different foods and it is one of the polyphenols that is not limited considerably in terms of bioavailability, evidenced by lower levels of peroxidation of plasma lipids. Consumption of quercetin in animal models as well as in humans correlated inversely to hypertension [171]. Quercetin appears to improve endothelial function in a NOS independent pathway [187]. Quercetin has anti-clotting abilities due to its ability to competitively bind plasminogen and also modulates plasmin concentration via urokinase plasminogen activator (uPA) modulation [188]. Quercetin’s anti-proliferative effect on vascular smooth muscle cells occurs primarily through inhibition of the JNK and AP-1 signaling pathways [185,189]. Quercetin has also been demonstrated to reduce ventricular hypertrophy, acting primarily to modulate Ang II [190,191]. Quercetin was also found to reduce cardiac myocyte apoptosis by preventing telomere shortening [139]. Thus, quercetin rich apples and onions can prove beneficial in protection of an aging cardiovascular system.

## 3. Functional Foods Rich in Polyphenols

### 3.1 Berry and Fruit Polyphenols—Anthocyanins, Flavonoids, Tannins

#### 3.1.1. Dietary Sources

The range of polyphenolic compounds that are found in berries is quite vast and encompass the flavonoids, namely, anthocyanins, flavanols and flavonols, condensed tannins (proanthocyanidins), hydrolysable tannins (ellagitannins and gallotannins), stilbenes and phenolic acids [192]. Berries are rich in anthocyanins and flavonoids, the most commonly found polyphenols in these fruits. The most widely consumed berries in the USA being blueberry, blackberry, raspberry, cranberry and strawberry while the less popular ones are acai and mulberry amongst others [39]. The anthocyanins are responsible for imparting the deep color to the berries and their concentration can range from 437.2 mg/100 g in raw chokeberries to 90.46 mg/100 g in blackberries [39]. The significant portion of the anthocyanin content is in the skin for most berries, but in a few like strawberries, they are contained in the flesh of the fruit. Grapes contain high amounts of polyphenols both in their skin and in their flesh. Also, polyphenols are found in grape seed extracts and in grape juice [193]. The polyphenols in grapes are phenolic acids, anthocyanins and flavonoids and their composition and content can vary depending on the location of grape cultivation [193]. The amount of resveratrol, which is one of the polyphenols in grapes, has been discussed in detail in the previous sections. Other dietary sources of anthocyanins include vegetables like red cabbage that contain 322 mg/100 g, whereas juices like pomegranate contain 15–252 mg/L and fruits like plum and grapefruit have lower content 2–25 mg/100 g and 5.9 mg/100 g respectively [194]. Other dietary sources of flavonols include apples (0.1–45 mg/100 g), plums (3.7–79 mg/100 g), and cherries (6.3–23 mg/100 g) to name a few [194]. Tannins are found in grape extracts and in red wine polyphenols (RWPs), in Indian blackberries (*Jamun*), plums, pomegranate *etc.* [192,195,196].

#### 3.1.2. Epidemiological Data and Clinical Studies on Berries and Fruit Polyphenols

Numerous human intervention studies have been undertaken to assess the relationship between the consumption of fruits and vegetables in reducing CVD related risks. This has been reviewed extensively elsewhere [197]. In brief, purple grape juice was found to be most potent in reducing platelet aggregation; a diet rich in flavonoids, for example intake of 50 mL/day pomegranate juice or 150 g/day mixed berry juice (bilberry, ligonberry, black currant, strawberry, and raspberry) significantly reduced blood pressure in hypertensives. Purple grape juice and pomegranate juice improved vascular function as well as improved blood lipid profiles [197]. In the Zutphen elderly study, an inverse correlation was found between consumption of flavonoid rich fruits and vegetables and mortality from coronary heart disease in elderly male subjects [98,198]. Another study on an elderly cohort, a subset of the CPSII nutrition study in the USA, also came to the same conclusions about fruits and vegetable consumption and CVD related mortality [199].

The FINRISK study was undertaken initially in 1972 to understand the reasons for the highest coronary heart disease (CHD) related death in Finnish men in the 1960s. The study went on until 2007 to analyze trends and monitor risk factors via a survey every 5 years, and to assess the association of lifestyle and dietary trends with mortality in Finland. The study reported that the intervention after the initial survey (in 1972 and 1977) resulted in a gradual decline in CHD related mortality in this population underscored by a reduction in blood pressure and improvement in blood lipid profiles. The reduction in mortality index over the 30 years (from 1977 to 2007) was attributed to increased awareness and changes in health behavior attributable to major changes in dietary patterns with increase in intake of vegetables and fruit and a change in type of fat consumed [200].

In another study in Finland, the Kuopio Ischemic Heart Disease Study (KIHD) analyzed whether a diet rich in fruits and vegetables is associated with a decreased risk of cardiovascular disorders. In this study the dietary intake of a population of men from Eastern Finland was analyzed by a qualitative assessment of nutritional intake using a questionnaire to record dietary intake over 4 days. The intake of fruits, berries and vegetables was 41% lower in the population that had the highest mortality at the 5 year follow up. The key findings of the study points to the increased occurrence of CVD and non-CVD related mortality in the group with the lowest consumption of fruits, berries and vegetables at the follow up after 12.8 years [201]. Further, the measures of cardiovascular health like serum HDL and cholesterol levels were also significantly higher in the group that consumed the least amount of fruits, berries and vegetables, and parameters for metabolic disorders such as insulin levels were increased as well [201].

The INTERHEART study involved participants from 52 countries to assess the relationship between diet and risk for acute myocardial infarction (AMI) globally. The diet of the subjects was classified as Oriental, Western or prudent with the Oriental diet being high in tofu and soy, the Western diet being high in fried food, salty snacks and meat while the prudent diet was high in fruits and vegetables. The study observed an inverse correlation between the risk for AMI and the prudent diet [202]. Interestingly, participants of the INTERHEART study were also analyzed in another report that studied the effect of gene-environment interactions and the predisposition to CVD. Four SNPs from chromosome 9p21 that had been previously identified as being associated with increased risk in MI were assessed in the INTERHEART participants and it was found that diet modified the risk associated with these SNPs, reducing it in individuals who consumed the prudent diet [203]. The same study also expanded to analyzing individuals from the FINRISK study, with the same conclusion that with increased consumption of vegetable and fruits, the risk of CVD, associated with alleles at chromosome 9p21, was significantly reduced [203].

#### 3.1.3. Mechanism of Action of Berry and Fruit Polyphenols—Cell and Animal Studies

The cardioprotective effects of berry and fruit polyphenols range from their anti-atherogenic and anti-inflammatory properties to their ability to modulate platelet aggregation and aid in recovery from ischemia reperfusion injury [39,197]. A few examples of cell and animal studies that aided evidence support of these cardioprotective properties are summarized here.

Apolipoprotein E deficient mice (Apo E) have an increased capacity for the intake of oxidized LDL into their macrophages, a hallmark of the early stages of atherosclerosis and foam cell formation [17]. Consumption of pomegranate byproduct by such mice resulted in 57% reduction in atherosclerotic lesion size. Further, in macrophages obtained from ApoE mice, the cellular lipid content was lowered by 53% and uptake of oxidized LDL was reduced by 19%, suggesting that pomegranate byproduct significantly attenuated the development of atherosclerosis [204]. ApoE deficient mice fed a diet rich in blueberries showed a recession in the symptoms of atherosclerosis by reducing the number of atherosclerotic lesions and importantly upregulating the synthesis of 4 key antioxidant enzymes in the liver and serum namely SOD 1 and 2, GSR, Thioredoxin (Tnxrd1) and serum Paraoxonase (PON1). Astonishingly, all these anti-atherosclerotic effects were observed without alterations in the lipid composition of serum; in fact both total and LDL cholesterol levels were higher in the blueberry diet group [205]. ApoE deficient mice fed a diet of added cholesterol and a low (4.75%) or high (9.5%) amount of dried plum powder showed no changes in serum cholesterol despite the added cholesterol in the diet, but demonstrated significant reduction in atherosclerotic lesions in the aortic arch and arterial tree [206]. Similarly, a diet supplemented with apple polyphenols and apple fiber as part of their diet also proved to be anti-atherosclerotic in ApoE mice by reducing size of atherosclerotic lesions, improving lipid profile and reducing oxidative stress parameters [207].

Polyphenolic grape extracts (PGE) are inhibitory toward platelet aggregation. These PGE, containing a high amount of gallic acid, inhibit thrombin receptor activating peptide (TRAP) induced platelet aggregation by reduced Ca^2+^ mobilization and an activation of platelet endothelial cell adhesion molecule-1 (PECAM-1), a molecule which is known to reduce thrombus formation [208,209]. Pomegranate juice is also inhibitory toward platelet activation by reduction in aggregation, H_2_O_2_ production and Ca^2+^ mobilization in addition to reduced TxA2 production in washed platelets stimulated with collagen or arachidonic acid [210]. Peroxisome Proliferator Activated Receptors (PPARs) are a family of nuclear receptors and transcription factors that are important in lipid metabolism, cell differentiation and have also been shown to play role in inflammation; agonists to PPARs have been useful in reducing cardiac pathologies due to hypertension and inflammation [211]. In the Dahl salt sensitive rat model, the animals that received dietary supplementation of grape seed powder showed an increase in cardiac PPAR activity and reduction in the PPAR agonist NFκB. The decreased NFκB activity also reduced the cardiac expression of the cytokine TNFα and the growth factor transforming growth factor β1 (TGFβ1) [212]. In a different study, the consumption of freeze dried tart cherries (1% w/w in the diet) by the Dahl-SS animals for 90 days, was also shown to enhance hepatic PPAR and improve the condition of hyperlipidemia and hyperinsulinemia in these animals [213].

In the SHRs fed an extract of ligonberry, cranberry and blackcurrant as part of their drinking fluid ad libitum for 8 weeks showed vastly improved vascular health and reduced inflammation. The mRNA expression of COX2, MCP-1, P-selectin, VCAM-1, and ACE were notably reduced in the cranberry and ligonberry extract fed animals [214]. The levels of circulating Ang II were significantly reduced, which otherwise leads to vasoconstriction. Flavanones can also modulate inflammatory process in atherosclerosis by reducing the binding of monocytes treated with Naringenin and Hesperitin, flavanones found primarily in citrus fruits, to TNFα activated endothelial cells [215].

Berry consumption may also be beneficial in reducing blood pressure. In a study utilizing the SHR model to analyze the effect of acetylcholine (Ach) induced vasorelaxation and phenylephrine induced vasoconstriction in animals fed a diet enriched with freeze dried blueberries (8% w/w with regular rat chow) for 7 weeks to assess the vasomotor tone in aortas of the animals. The study demonstrated that the Ach mediated vascular smooth relaxation was increased due to the blueberry diet, amplified when the NO pathway was blocked using l-NMMA and attenuated when the COX pathway was eliminated using Mefenamic acid a COX inhibitor, suggesting these berries affect the NO and COX pathway stimulated by Ach [216]. In the stroke prone SHRs, consumption of freeze dried blueberries (3% w/w) for 8 weeks reduced blood pressure by 19% by week 4 and 30% by week 6 relative to controls [217]. This reduction in blood pressure may in part be due to the inhibition of ACE activity and lowered Ang II levels in the plasma [218]. Reduction of blood pressure in SHR was also accomplished by intake of raspberry ethyl extracts; this was accompanied by increase in SOD and serum NO and lowered MDA and plasma ET-1 [219].

In a study of recovery from ischemia, Sprague Dawley rats were fed with an extract made from either grape skin or grape flesh (2.5 mg/kg/day) for 30 days. The hearts were isolated and ischemia induced for 30 min followed by a 2 h reperfusion. Post ischemic function was assessed by blood flow through the coronary artery, myocardial infarct size and estimation of ROS by measuring malonaldehyde (MDA) in the heart. The data showed that both grape skin and flesh were equally cardioprotective as evidenced by improved recovery from ischemia, smaller infarct size and reduced MDA content in the heart as compared to the control group [220]. Blueberry supplementation in rats that underwent coronary ligation induced myocardial infarction showed a 22% improvement in survival rates as well as reduction in the expansion of myocardial infarcts [221,222]. Mechanistically, this might be achieved by stimulating the synthesis of NOS via the Akt pathway, as was the case in chick embryonic cardiomyocytes stimulated with grape seed extract, thereby protecting from I/R induced cell death [223].

#### 3.1.4. Berries and Fruits in Cardiovascular Aging

Berries contain a broad range of phenolics with strong antioxidant potential. Anthocyanins (billberries, blackberries, crowberries), hydroxycinnamic acid (currants, blueberries and sweet cherries), flavonol (blueberries), flavan-3-ol (red raspberries), caffeic acid, gallic acid, quercetin, rutin and naringenin (mulberry leaves) are some of the better studied phenolics with antioxidant function [224,225,226,227]. Pomegranates have also been demonstrated to have anti thrombolytic activity and a strong antioxidant ability [228]. Also, pomegranate juice improved antioxidant function in elderly subjects [229]. Antioxidant defenses stimulated by anthocyanins improved endothelial function by NO dependent relaxation of arteries. Anthocyanins are anti-thrombolytic by virtue of inhibiting thrombin receptor-activating peptide-induced platelet aggregation. They are anti-inflammatory and inhibit NFκB and CD40 signaling pathways. Anthocyanins reduce VEGF secretion and down regulate p38 MAPK and c-Jun *N*-terminal kinase pathways, thereby impacting proliferation and migration of VSMCs [194,230]. Hydroxycinnamic acid has been shown to be a strong free radical scavenging polyphenol [231]. Herperdin, the flavonone found in citrus fruits, improved the antioxidant enzymes in aged rat hearts by upregulation of SOD, CAT, and Gpx amongst others, reduced the levels of MDA and increased the level of Nrf-2 [232]. In summary, berries and fruits have several synergistically functioning polyphenols that offer significant cardioprotective functions. The Zutphen elderly study, and other studies on cohorts of elderly subjects have undoubtedly shown the importance of a diet rich in fruits and vegetables as an important parameter for healthy aging and reduced CVD risk [98,198,199].

### 3.2 Olive Oil Polyphenols—Hydroxytyrosol and Oleuropein

#### 3.2.1. Dietary Sources

Previously; the beneficial health effects of olive oil were attributed primarily to its high monounsaturated fatty acid (MUFA) content; mainly in the form of oleic acid; MUFA content being 56%–84% of total fatty acid content [233]. When the antioxidant ability of olive oil was demonstrated in a variety of studies; it brought to limelight the most representative phenolics in extra virgin olive oil namely hydroxytyrosol and oleuropein [233,234]. There is a clear distinction among types of olive oil in terms of polyphenolic content; since extra virgin olive oil has a greater concentration of polyphenolic compounds than refined olive oil [233,235]. There are a variety of factors which affect the quality of the oil; primarily its oxidative stability which is in turn dependent on the MUFA/PUFA ratio; the higher the ratio; the better the oil; however, as the fruit ripens; this ratio falls [236]. Furthermore, the riper the olive; the lower the quantity of phenolics in the oil [237]. Also; some authors have argued that the oxidative stability of the oil is dependent on the phenolic content; therefore, a ripening index (RI) must be determined for each olive cultivar to extract oil with maximum stability in terms of high MUFA/PUFA ratio as well as higher phenolic content [238].

#### 3.2.2. Epidemiological Data and Clinical Studies on Olive Oil

Large majority of studies attribute the beneficial health effects of the Mediterranean diet to olive oil because of its MUFA content. MUFAs are reported to improve heart disease risk factors by lowering total cholesterol levels as well as LDL cholesterol [239,240]. However, minor constituents of olive oil are also suggested to play a larger role in cardiovascular health, see review [241]. In fact, extra virgin olive oil has cholesterol lowering effects that are independent from the fatty acid content of the oil. For instance, diets enriched with virgin olive oil having a polyphenolic composition of 34.3 ± 1.5 mg/100 g resulted in a 7.3% reduction of LDL cholesterol in volunteers compared to baseline measurements [242]. Further, extra virgin olive oil had a greater antioxidant effect on the *in vitro* oxidation of LDL induced via peroxyl radicals and metals than refined olive oil [235]. Healthy male volunteers were administered a daily dose of olive oil containing any of the three varying levels of polyphenol content: low, medium or high. Volunteers with any intervention demonstrated increased HDL levels, decreased total cholesterol:HDL ratio and triglyceride levels, as well as an improved oxidized:reduced glutathione ratio. Interventions, excluding the low polyphenol olive oil, decreased LDL:HDL ratio and high polyphenol olive oil had reduced hydroxyl fatty acids and circulating oxLDL levels. Olive oil with increasing phenolic composition resulted in increasing HDL levels, decreasing total cholesterol:HDL ratio and oxidative biomarkers [243,244]. Similar studies indicate the beneficial effects of virgin olive oil compared to refined in terms of lipid profiles of healthy subjects [239,245,246]. Comparable doses of extra virgin olive oil to raw intake in some Spanish regions resulted in lower oxLDL and lipid peroxide levels and higher glutathione peroxidase activity in males with stable CHD. There is also evidence of anti-hypertensive effects of virgin olive oil since these males with CHD also had reduced systolic blood pressure [247]. In addition, extra virgin olive oil reduced inflammatory markers, TXB2 and LTB4, in 12 healthy participants consuming 150 g of mashed potatoes with 50mL of olive oil [248].

Antioxidant properties of the polyphenolic compounds found in olive oil were corroborated by Visioli, Bellomo, & Galli (1998) [234]. Radical scavenging activity, including that of superoxide anions, as well as protecting against hyperchlorite damage and inactivation of catalase were witnessed with hydroxtyrosol and oleuropein [234,249]. Importantly, there is evidence indicating that these major polyphenols, including tyrosol, have a high bioavailability in humans [240].

#### 3.2.3. Mechanism of Action of Olive Oil—Cell and Animal Studies

The protective effects of the olive oil polyphenols, hydroxytyrosol and oleuropein, are reported in *in vitro* models of LDL oxidation. Hydroxytryosol inhibits the increase of lipid peroxidation markers, F2-isoprostanes and TBARS, as well as production of superoxide anion and H_2_O_2_, in addition to, preserving total glutathione content, GSR and GPx activity and transcript levels [250,251,252]. Oleuropein inhibited oxidation of lipoproteins, generation of superoxide anion and H_2_O_2_ as well, and reversed the decreased glutathione content, GSR and GPx activity observed with LDL oxidation [253].

Olive oil polyphenols have a potential role in improving the pathophysiology of atherosclerosis. For example, olive oil intervention improves endothelial function and reduces inflammation parameters, thereby, aiding in the treatment of atherosclerosis patients [254]. Experimental studies involving rabbits subjected to an atherogenic diet enriched with saturated fatty acids display hyperactivity of platelets, thrombogenicity and poor lipid profiles. Supplementation with olive oil improved the outcome of atherosclerosis via improving the lipid profile, reducing platelet hyperactivity and endothelial thrombogenicity and reducing the severity of endothelium and vascular wall lesions [255]. Similarly, rabbits fed a diet supplemented with hydroxytyrosol following an atherogenic diet had an improved lipid profile and a diminished total cross-sectional area and that of the intima layer of the aortic arch was observed [256]. Further, platelet aggregation is inhibited via the phenol component of olive oil [257]. Similar investigations have demonstrated the antioxidant and anti-atherogenic effects of olive oil polyphenols on the cardiovascular system [258]. However, it should be noted that hydroxytyrosol does not enhance eNOS or NO bioavailability in healthy circumstances. It is possible that hydroxytyrosol could preserve eNOS function indirectly under pro-inflammatory conditions only [259]. Inflammatory angiogenesis has a role in the progression of atherosclerosis, which is markedly reduced with *in vitro* treatment of olive oil polyphenols. The antioxidant effects of olive oil polyphenols are attributed to the inhibition of COX-2 and MMP-9 which are regulated by redox-sensitive signaling pathways and typically promote angiogenesis [260]. Extra virgin olive oil reduced cell surface expression of VCAM-1 and ICAM-1 in an *in vitro* model of inflammation [261,262]. Oleuropein and hydroxtyrosol reduced monocyte adhesion to the endothelial primarily due to the inhibition of VCAM-1 mRNA and protein expression. Further, transcriptional activation of the VCAM-1 gene via NF-κB and AP-1 was inhibited by the olive oil polyphenols screened [261,263]. Similar reductions were seen in the expression of adhesion molecules: *E*-selectin and ICAM [263]. Oleuropein demonstrates anti-proliferative properties on VSMCs by blocking cells in G1 to S phase via inhibition of ERK1/2 activation [264].

Oxidative stress is involved in myocardial damages associated with I/R, which suggests that the antioxidant capabilities of olive oil polyphenols might have a potential protective effect in the pathophysiology of I/R injury. For example, treatment with oleuropein prior to I/R induction in rat hearts resulted in a reduction in release of oxidize glutathione, a marker of the heart’s exposure to oxidative stress. Additionally, oleuropein decreased creatine kinase activity, another measure of myocardial damage [265].

Polyphenols found in olive oil, particularly extra virgin olive oil, are recognized as having an important role in the protection of cardiovascular ailments including improvements in lipid profiles within clinical and experimental studies. It is important to note that these cardiovascular health benefits are not only attributed to the MUFA content of olive oil but to these polyphenols as well.

#### 3.2.4. Olive Oil in Cardiovascular Aging

Olive oil is rich in MUFAs and over 30 different phenolic compounds, which together, make this potent super food likely to be a key player in lowered incidence of atherosclerosis and CVD in aging Mediterranean populations. A study on elderly people consuming the Mediterranean diet, reported that the proinflammatory markers NF-κB, MCP-1, TNF-α and IL-6 as well as atherogenic marker MMP-9 was reduced in PBMC from these subjects [266]. Additionally, the bioavailability of olive oil phenolics remains high after ingestion [267]. The predominant phenolic compounds are α-tocopherol, hydroxytyrosol, tyrosol, and oleuropein [268]. All four are potent antioxidants, with α-tocopherol and hydroxytyrosol being identified to have the greatest antioxidant scavenging potential [269]. Additionally, tyrosol has been implicated in stimulating antioxidant defenses by modulating the phosphorylation of Akt, eNOS and SIRT1 [270]. In a study on the senescence-accelerated mouse-prone 8 (SAMP8) mice, an animal model employed to understand the molecular mechanisms of age related changes, olive oil ingestion reduced cardiac oxidative stress by inducing Nrf2 and Nrf2 dependent genes as well as increased PON1 activity and SIRT1 mRNA [271]. The regulation of Akt and consequently the FOXO proteins as well as control of SIRT1 likely regulates cell viability of cardiomyocytes in the heart as well. In modulating the anti-inflammatory response, IL-1B levels are decreased by oleuropin [272] and COX1 and COX2 have been shown to be inhibited by another phenolic, oleocanthal in a dose dependent manner [273]. Extra virgin olive oil consumption (25 mL/day for 3 weeks) showed reduction in anti-inflammatory properties as a function of aging in healthy elderly subjects by modulating PON1 and decreasing age related atherogenic changes [274]. Proliferation of VSMCs in the aging arterial laminae are likely modulated by selective NO level based Akt inactivation by hydroxytyrosol [275]. Phenolics in olive oil have also been shown to modulate clotting, thereby adding to their cardioprotective value in an aging cardiovascular system [257,276]. A staple of Mediterranean communities, evidence suggests that olive oil clearly contributes considerably to a robust cardiovascular system.

## 4. The Potential and Limitations of Polyphenols in Treatment of Human Heart Disease

Polyphenols can exert beneficial effects via their antioxidant capabilities yet interventions with antioxidants in clinical trials have been unsuccessful in the prevention and treatment of cardiovascular diseases. In particular, β-carotene, an antioxidant most commonly found in carrots, pumpkin, sweet potato, and others showed promise in experimental studies but failed in human trials. After 12 years of treatment, no significant benefit or harm of β-carotene treatment on myocardial infarction, stroke or cardiovascular death was determined [277,278]. Clinical trials of polyphenols as supplements for cardiovascular disease have also yielded inconsistent results, both positive and ineffectual. The following studies are a few examples of studies that demonstrated positive effects of polyphenol supplementation in heart disease patients. Consumption of resveratrol-rich grape supplement for 1 year improved inflammatory and fibrinolytic status of patients undergoing primary prevention of cardiovascular disease [59]. Additionally, blood pressure of 67 men at high cardiovascular risk was attenuated following a 4 week intervention of dealcoholized red wine [279]. Consumption of 7 mL/kg/day of purple grape juice inhibited platelet aggregation in 20 healthy subjects as well as increased NO production, α-tocopherol levels and decreased superoxide release [280]. Participants in a clinical study investigating the effects of freeze-dried blueberry beverage on cardiovascular risk factors showed positive results. These participants presenting with metabolic syndrome had a decrease in blood pressure with the 8 week blueberry intervention [281]. Positive effects on brachial artery vasomotor function were seen after consumption of black tea after 2 h and 4 weeks in 66 patients with coronary artery disease [282]. EGCG supplementation at 400mg reduced diastolic blood pressure in 46 overweight/obese males [283]. Additionally, throughout this review, various examples of other clinical as well as epidemiological studies have been highlighted that have emphasized the significance and correlation of diets rich in polyphenols and better cardiovascular health. However, some clinical trials have not had success with polyphenol and functional food intervention. For instance, capsules containing 800 mg polyphenols derived from either wine grape mix or grape seeds had no major impact on flow-mediated dilation in 35 healthy males [284]. Similarly, intervention with polyphenol-rich or polyphenol-poor apples did not affect flow-mediated dilation or other cardiovascular disease risk factors of 30 hypercholesterolemic volunteers [285]. Finally, biomarkers of cardiovascular disease risk were unchanged with a daily 3 week intervention of six capsules containing green tea extracts [286]. Similarly, green and black tea supplementation had no effect on cardiovascular disease risk parameters [287,288].

Further adverse effects of these compounds have also been indicated in a few studies. Though the studies reported are not necessarily all pertaining to cardiovascular conditions, it is important to note that polyphenols have been shown to be disadvantageous in certain circumstances related to interactions with other substances, pro-oxidant activity, toxicity and tumorigenesis [289]. Polyphenols can influence availability of a number of compounds, by binding to and forming complexes with proteins, and metal cations affecting their absorption [290]. For instance, the bioavailability of iron within humans was shown to be affected by tea, a major source of polyphenols [291,292]. However, two recent studies concluded that tea consumption does not influence iron status in populations where iron intake is adequate [293,294]. Polyphenols can have pro-oxidant effects, which normally aid in the plant’s defense system. In the presence of oxygen and transition metals, such as copper and iron, redox cycling of polyphenols is initiated to generate ROS, leading to cellular injury including DNA damage [295]. Toxicity of polyphenols, particularly EGCG, was determined at a concentration of 2000 mg/kg/day in rats of which lethality was the measure of toxicity. In the same study, it was determined that doses up to 500 mg/kg/day for 13 weeks was not toxic in these animals [296]. Tumorigenesis has also been documented *in vitro* and *in vivo* models employing treatments with polyphenols. For example, carcinogen-exposed rats treated with 0.1% green tea catechins had an increase in average tumor size compared to controls. Interestingly, supplementation with 1% green tea catechins did not affect tumor size. Neither dose of green tea catechins contributed to incidence nor multiplicity of tumors in these animals [297]. Similarly to the animal study, cell proliferation in a cell culture model was stimulated with a lower concentration of quercetin yet a higher concentration decreased cell proliferation [298]. Thus, dosage of polyphenols is important to minimize harmful effects of their use.

Therefore, consideration for the bioavailability of these compounds, dosage parameters, and the interaction of polyphenols and other bioactive compounds in functional foods is important and may contribute to the effectiveness of interventions described in the clinic [299]. Bioavailability of these compounds as well as pharmacological properties and kinetics of absorption need to be further evaluated to improve our comprehension of their behavior within the body, and to aid in the investigation of intervention studies. More stable analogs with similar bioactivities but better bioavailability are being designed with the goal of developing functional nutraceuticals and to minimize harmful effects [300].

## 5. Conclusions

**Figure 1:**
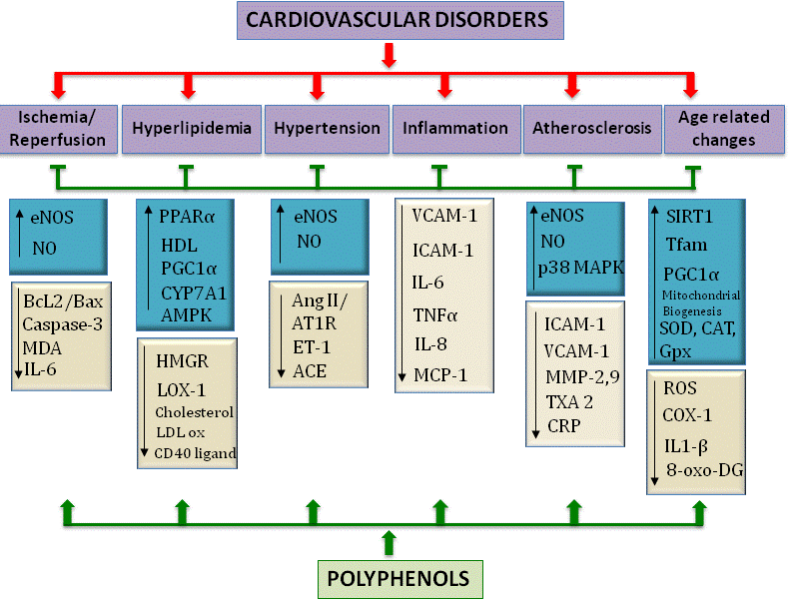
Mechanisms of protection by polyphenols in the cardiovascular system, in disease and in aging.

A number of epidemiological trends and clinical studies support the notion of a diet rich in fruits and vegetables being correlated with reduced cardiovascular complications and mortality. Polyphenol rich diets have been associated with reducing CVD risk thereby promoting optimal aging. The literature reviewed here validates that treatment of cells and animal models with polyphenols counteracts the burden of oxidative stress and influences signaling pathways to diminish the risk associated with cardiovascular diseases, and endorses their therapeutic efficacy in functioning as anti-aging molecules (Figure 1). These findings are sufficiently corroborated by longitudinal studies on human subjects, with some of the studies being reported in our review. It may not be single polyphenols but a combination found in a given food that may be responsible for the health benefit seen across populations. As Wersching (2011) points out, the direct interplay between nutrients in whole fruits and vegetables may be more important than the unique nutrients on their own in the reduction of risk in cardiovascular disease [301]. Further studies on bioavailability and kinetics of absorption need to be undertaken with the goal to find the combinatorial mix that acts synergistically upon the various targets, to then as a whole reduce the economic burden of cardiovascular disease and to promote healthy aging.

## Abbreviations

ROS: reactive oxygen species
CVD: cardiovascular disease
CHD: coronary heart disease
CAD: coronary artery disease
AMI: acute myocardial infarction
EGCG: epigallocatechin gallate
SOD: super oxide dismutase
GPx: glutathione peroxidase
GST: glutathione *S*-transferase
GSR: glutathione reductase
NQO1-NAD(P)H: quinone oxidoreductase 1
HO-1: hemoxygenase-1
eNOS: endothelial nitric oxide synthase
MMPs: metalloproteinases
VCAM-1: vascular endothelial adhesion molecule-1
ICAM-1: intercellular cell adhesion molecule-1
MCP-1: monocyte chemoattractant protein-1
TNF α: tumor necrosis factor α
PDGF: platelet-derived growth factor
ACE: angiotensin converting enzyme
Ang II: angiotensin II
ET-1: endothelin-1
LDL: low density lipoprotein
HDL: high density lipoprotein
VSMC: vascular smooth muscle cell
PPAR: peroxisome proliferator activated receptor
PGC: peroxisome proliferator activated receptor coactivator
SHR: spontaneously hypertensive rat
WKY: Wistar Kyoto rat
SHR-SP: SHR stroke prone
DSS: Dahl salt sensitive rat
ApoE: apolipoprotein E deficient mice
LDL-KO: LDL receptor knockout
